# Understanding Delayed T-Cell Priming, Lung Recruitment, and Airway Luminal T-Cell Responses in Host Defense against Pulmonary Tuberculosis

**DOI:** 10.1155/2012/628293

**Published:** 2012-04-01

**Authors:** Christopher R. Shaler, Carly Horvath, Rocky Lai, Zhou Xing

**Affiliations:** McMaster Immunology Research Centre; Department of Pathology and Molecular Medicine, McMaster University, Hamilton, ON, Canada L8S4K1

## Abstract

*Mycobacterium tuberculosis* (*M.tb*), the causative bacterium of pulmonary tuberculosis (TB), is a serious global health concern. Central to *M.tb* effective immune avoidance is its ability to modulate the early innate inflammatory response and prevent the establishment of adaptive T-cell immunity for nearly three weeks. When compared with other intracellular bacterial lung pathogens, such as *Legionella pneumophila*, or even closely related mycobacterial species such as *M. smegmatis*, this delay is astonishing. Customarily, the alveolar macrophage (AM) acts as a sentinel, detecting and alerting surrounding cells to the presence of an invader. However, in the case of *M.tb,* this may be impaired, thus delaying the recruitment of antigen-presenting cells (APCs) to the lung. Upon uptake by APC populations, *M.tb* is able to subvert and delay the processing of antigen, MHC class II loading, and the priming of effector T cell populations. This delay ultimately results in the deferred recruitment of effector T cells to not only the lung interstitium but also the airway lumen. Therefore, it is of upmost importance to dissect the mechanisms that contribute to the delayed onset of immune responses following *M.tb* infection. Such knowledge will help design the most effective vaccination strategies against pulmonary TB.

## 1. Introduction: Current Challenges and Global Impact

**Figure 1:**
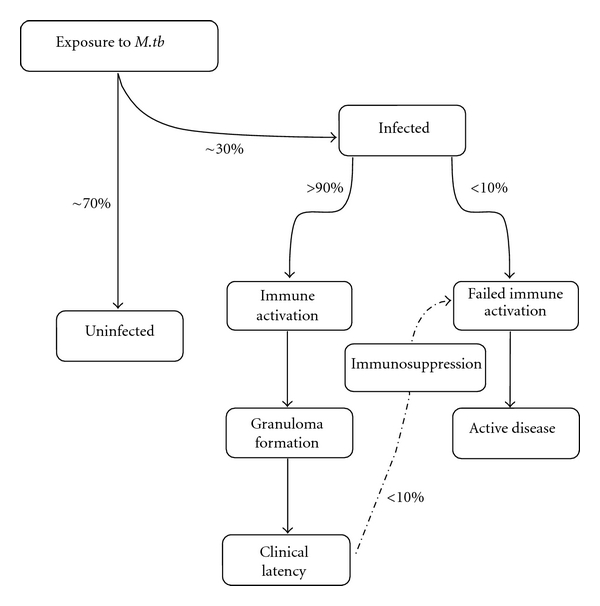
Flow chart of TB disease progression and major events leading to protection. Major steps are outlined for the progression of and infected or uninfected hosts from the point of exposure to development of active disease or clinical latency (protection). The relative percentage of individuals to progress between steps is shown beside the appropriate progression line.

Despite extensive vaccine coverage in endemic areas, pulmonary tuberculosis (TB) remains one of the top three infectious causes of death worldwide [1]. With an alarming 9 million new cases annually, it is estimated that one third of the world's population is latently infected with *Mycobacterium tuberculosis *(*M.tb*), the causative bacterium of TB [1]. Following primary infection, greater than 90% of infected individuals enter into an asymptomatic latency period, showing no clinical sign of disease (Figure 1). The ability of the host to control *M.tb* is accomplished through bacterial restriction and segregation, rather than clearance. *M.tb*, a facultative intracellular pathogen, is spread person-to-person through infected aerosols generated by coughing or sneezing. Once deposited in the airways, *M.tb* primarily infects the alveolar macrophage, the resident macrophage of the airway lumen [2, 3]. *M.tb *has a transmission rate of 30% or less but the relative susceptibility of an exposed individual to infection is determined by a number of factors including the living conditions, contact time with infected individuals, and immune status [4–7]. It is estimated that under the correct conditions a single bacillus could establish a successful infection [2]. The fact that *M.tb* is spread via aerosols, and can be infectious in low numbers makes it a major health concern in regions such as those commonly seen in developing world with high population densities, poor living conditions, and immune compromising diseases.

While many infections remain asymptomatic, the sheer number of infected individuals makes TB the number one bacterial killer worldwide, responsible for nearly 2 million deaths annually [1]. The majority of TB-related deaths are seen in the developing world where infected individuals cannot afford the lengthy antibiotic regime required to treat *M.tb *[1]. Compounding the problem, the only licensed TB vaccine, Bacillus Calmette-Guerin (BCG), has shown highly variable efficacy (0–80%) [8]. Even with the highest efficacy, BCG is only effective in limiting severe disseminated forms of TB in children, not preventing lung disease or providing sterile immunity [8]. Moreover, the usefulness of BCG is further limited as protective immunity typically wanes by adolescence [9] and cannot be boosted by repeated BCG vaccination [10]. Further compounding the problem, many of the regions with the highest incidence of TB also coincide with those endemic to HIV-AIDS [11]. The ability of HIV-AIDS to dramatically suppress cellular immunity has made coinfections with *M.tb* particularly deadly. Of the estimated 2 million deaths by TB per year, approximately 400,000, nearly one quarter, are of HIV-TB-coinfected individuals, highlighting the significance of this deadly coinfection [11].

As one of the most persistent global health concerns, the success of *M.tb* as a human pathogen can be attributed to its ability to parasitize the host-pathogen microenvironment. Studies of ancient DNA and skeletal remaining have traced the coevolution of *M.tb* and prehuman lineages for nearly 3 million years [12–16]. As such, *M.tb* has evolved multiple mechanisms to evade, elude, and subvert the host immune system. For instance, compared to many other respiratory pathogens, *M.tb* infection retards adaptive T-cell activation by eliciting much delayed T-cell priming and lung recruitment [17, 18]. Temporally, *M.tb* targets both early immune initiation as well as chronic bacterial control preventing the host from ever achieving sterile immunity. While much research has been done to understand the various ways *M.tb *suppresses established immunity, little progress has been made in understanding the mechanisms underlying delayed early adaptive immune activation.

To this end, it has been proposed the impairment in adaptive immune activation may be attributed to a combination of underlying defects in the immune initiation cascade. Specifically, the limited availability of antigen due to the slow replication rate of *M.tb* is thought to account for a weak early inflammatory response, delaying the recruitment of innate immune cells into the lung. The impaired entry of immune cells into the lung coupled with active immune suppression driven by *M.tb* are the major mechanisms thought to delay the migration of antigen-presenting cells (APC) to the lymph node. Puzzling and poorly understood, the contributions of both host and pathogen to the relative delay in T-cell activation still remain largely unresolved.

## 2. Initiation of Innate Immunity in the Lung Following *M.tb* Infection

### 2.1. Infection

*M.tb* is spread through aerosols generated by an infected individual [19]. Coughing or sneezing is the primary method of transmission, and persons with active disease are highly contagious [11]. Infected aerosols are taken into the lung and deposited in the alveolar space, where *M.tb* is actively taken up by the resident alveolar macrophage (AM) via phagocytosis [20]. Once engulfed by the macrophage, *M.tb* becomes highly resistant to clearance. This is achieved by evading immune detection and elimination through a variety of immune evasion strategies, including blocking phagolysosome fusion and detoxifying oxygen and nitrogen radicals [21]. Although the primary cell type to be infected is the AM, *M.tb *can also actively infect and replicate within recruited neutrophils [22], dendritic cells [23], and alveolar type II epithelial cells [24].

### 2.2. Innate Recognition

 Upon entering the airway lumen, *M.tb* is thought to “silently” enter the resident AM. Infection of the AM occurs through receptor-mediated phagocytosis. Utilizing the complement receptors (CR3 and CR4), the mannose binding receptor, surfactant molecules, and DC-SIGN, *M.tb* rapidly facilitates its uptake by the AM [25–27]. Upon entry, recognition of *M.tb *is mediated through the engagement of pattern recognition receptors (PRRs). While the toll-like receptors (TLRs), specifically TLR-2, 4, and 9, have long been recognized as the primary PRRs required for the detection of *M.tb *[28, 29], recently a member of the NOD family of receptors, NOD2, has been shown to play a critical role in the intracellular recognition and activation by the *M.tb*-infected macrophage [30]. Typically, the AM acts as a sentinel, detecting and alerting surrounding cells to the presence of an invader. However, in the case of *M.tb*, this function is thought to be impaired. In particular, *M.tb* has been shown to uniquely engage the mannose receptor (MR) of responding macrophages. A major cell wall component of *M.tb, *lipoarabinomannan (LAM), is alternatively capped with mannose, which signals through the MR, inducing an anti-inflammatory program; impairing the secretion of both proinflammatory cytokines (TNF-*α* and IL-1*β*) and chemokines (MCP-1 & IP-10) [27, 31], thereby deferring the recruitment of innate immune cells to the lung. Interestingly, *M.tb* appears to simultaneously induce both pro- and anti-inflammatory effects as it has recently been shown that *M.tb* interacts with the airway epithelium to induce the production of MMP-9, a mechanism to attract macrophages to the site of infection facilitating its own propagation [32].

### 2.3. Recruitment of Antigen Presenting Cells (APCs) to the Lung

**Figure 2:**
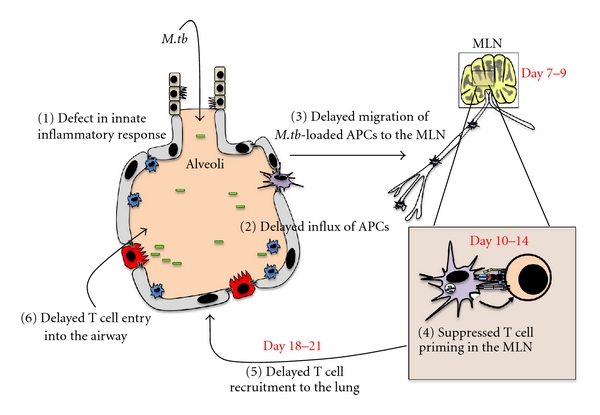
Illustration of the speculated major immunologic setbacks seen in the early course of pulmonary *M.tb* infection. The major defects are numbered in the diagram according to the sequence of events. APC: antigen-presenting cells; MLN: mediastinal lymph nodes.

 The entry of responding immune cells into the lung involves cell migration across the vascular endothelium and the airway epithelium that separates the lung interstitium and airway luminal space. Although not much has been studied in the context of TB, it is understood that crossing the endothelium requires appropriate activation, involving tight junction modification and the expression of addressin molecules on the luminal surface. These processes are significantly inducible by inflammatory cytokines such as TNF-*α* and IL-1*β* [33–36]. Preliminary findings from our group suggest that there is a minimum recruitment of innate immune cells to both the lung interstitium and airway lumen for first 5 days postmycobacterial infection, which is associated with a lack of both TNF and IL-1*β* induction in the lung during this time period [unpublished data]. It is thus our belief that the absence of an early innate inflammatory response in the lung represents an important mechanism delaying the upregulation of chemotactic and adhesion signals and the subsequent recruitment of innate immune cells to the lung, which in turn delays T-cell priming in the draining lymphoid tissues (Figure 2).

### 2.4. Mycobacterial Antigen Acquisition

 Under noninflammatory conditions, immune surveillance of the airway lumen is passive and mediated primarily by a limited number of intraepithelial dendritic cells (DCs) [37–39]. However, upon the initiation of an inflammatory response, there is a rapid recruitment of DCs to the various lung compartments [37]. The precise timing of these inflammatory events is not fully understood in the context of *M.tb* infection. However, as discussed above, it is believed that the major influx of APCs into the lung is delayed for the first 5–7 days following infection [40] and the trafficking of immune cells from the vasculature to the airway lumen is a two-step process. The majority of DCs first exit the vasculature into the interstitium and then migrate through the interstitial matrix and reach the alveolar epithelium [34, 41]. Rather than fully entering the airway lumen, the majority of recruited DCs interdigitate into the epithelial wall, extending their dendrites into the luminal space where they acquire antigen or microbial organisms while maintaining access to the collecting lymphatics located in the interstitium [37, 42].

While TNF has many functions, it is considered central to the appropriate control of an *M.tb* infection. During the initial stages, TNF acts primarily as an alarm cytokine alerting surrounding cells to the presence of an infection. It is believed that AM-derived TNF commences the recruitment of innate immune cells by activating the type II alveolar epithelial cells (AEII). This initiates the production of chemokines such as MCP-1, upregulates critical adhesion molecules, and reduces tight junction adhesion [43–45]. Following *M.tb* infection, it is unclear how the vascular endothelium becomes activated. However, given their geographic location and based on the knowledge from other models, it is plausible that the AEII relay the inflammatory signal from the airway to the interstitium, thereby activating the endothelium. AEII are central to the initial recruitment of APCs to the lung, functioning as the “gatekeepers” of the airway lumen controlling the production of chemokines such as MCP-1 and regulating the expression of addressin molecules [38, 46]. The early mobilization of APCs to the lung is critical to the timely control of an *M.tb* infection as it has been shown that in the absence of MCP-1's receptor, CCR2, APC recruitment to the lung is significantly delayed, impairing the downstream adaptive immune activation and bacterial control [47]. Upon entering the lung, it is currently unknown which subsets of DCs are primarily responsible for acquiring and transporting antigen or mycobacteria to the draining lymph node. Further complicating our understanding, *M.tb* actively infects macrophage, DC, and neutrophil populations, all of which have been shown to be capable of transporting antigen to the MLN [22, 48–50].

## 3. Initiation of Adaptive T-Cell Activation in the Mediastinal Lymph Node (MLN)

While much controversy surrounds the generation of adaptive immune responses following *M.tb*, it is now widely accepted that the earliest antigen-specific T-cells are not observed for at least 10 days postinfection, appearing first in the MLNs [51] (Figure 2). This delay is highly significant when compared to lung infection by other intracellular pathogens such as influenza or *Legionella pneumophila, *where adaptive responses are seen as early as 2-3 days in the MLN [52, 53] and in the lung 6-7 days postinfection [18, 54, 55]. This may suggest that such delay is due to insufficient bacteria or bacterial products in the lymph node required for T-cell priming [40]. Much controversy surrounds the precise arrival of *M.tb* to the MLN as some groups have identified *M.tb* in the MLN as early as 4 hrs postinfection, while others cannot detect *M.tb* for 7–9 days postinfection [40, 56]. Nevertheless, it is now well established that viable *M.tb *resides within the MLN several days prior to the emergence of the effector T-cells [23, 40]. We also detected viable mycobacteria in the MLN within a few days post-infection [unpublished data]. As *M.tb* is slowgrowing, it is possible that the antigenic threshold may not be reached in the MLN until days after mycobacterial arrival. Alternatively, the delayed T-cell priming could be due to insufficient APC activation and/or active suppression by *M.tb* of T-cell priming. Indeed, considered central to this delay, *M.tb* directly infects DCs, impairing both their capacity to migrate to the lymph node and activate naïve T-cells [23, 57].

### 3.1. Migration of DCs to the MLN

 The ability of antigen-loaded DCs to home to the MLN is largely due to the upregulated expression of chemokine receptor CCR7. CCR7 expression allows activated DCs to rapidly migrate towards the CCL19/21 chemokine gradients generated by the MLN [39]. Khader et al. [58] have demonstrated the dependence of IL-12p40 for the expression of CCR7 by *M.tb*-infected DCs. To this end, impairing the production of IL-12p40 was found to delay the migration of infected DCs to the MLN, thus deferring T-cell priming by several days [58]. Furthermore, it has also been demonstrated that *M.tb* directly induces a splice variant of the IL-12 receptor, significantly enhancing the responsiveness of infected DCs to IL-12p40, augmenting the migratory capacity of these populations [59]. It is proposed that *M.tb* may utilize the induction of this high efficacy receptor to facilitate its dissemination away from the lung in the manner similar to a Trojan horse [59]. These data suggest that that the delay in T-cell priming is a result of impaired DC functionality in the lymph node rather than the impaired migration of DCs into this compartment [60, 61].

### 3.2. Migrating APC Subsets

 While the intraepithelial DC may be the primary APC sampling mycobacteria or mycobacterial antigen in the airway lumen, it has been proposed that the AM may egress from the airway lumen into the interstitium, accessing the collecting lymphatics and transporting *M.tb* to the MLN [49]. Within the lung, it is difficult to appropriately classify the APC populations based on a single cell surface marker. To this end, the expression of conventional markers such as CD11c must be used in conjunction with other makers such as MHC class II expression to denote activated DC populations [39, 62]. In recent years, there has been a movement to classify the migratory potential of specific DC cell populations in the lung based on cell surface marker expression. While far from resolved, two distinct DC populations have been identified based on their potential to migrate to the MLN. The expression of CD103^+^ (CD11c^hi^  CD11b^−^ MHC-II^hi^  CD103^+^) has recently been shown to be important in transporting apoptotic bodies and mediating Ag cross-presentation to CD8^+^ T-cells during many viral infections [63]. The expression of CD11b^+^ (CD11c^hi^ CD11b^hi^ MHC-II^hi^  CD103^−^) has been shown to be key to the delivery of the majority of viable mycobacteria to the MLN [23]. From our preliminary studies, we have seen a surge in both DC subsets in the MLN following pulmonary mycobacterial infection [unpublished data]. As described above, complement represents one of the major mechanisms responsible for the uptake of mycobacteria by recruited APC populations. As such, it can be considered that the expression of both CD11b and CD11c, components of complement receptors 3 and 4, respectively, may allow for more efficient uptake of mycobacteria by these APC subsets. An enhanced capacity to uptake mycobacteria may provide a plausible, yet unconfirmed, explanation for why CD11c^+^CD11b^+^ DCs represent the dominant APC population during *M.tb* infection. The relevance of these DC populations with regard to the efficiency of antigen presentation and subsequent T-cell priming is still currently unknown in the context of *M.tb* infection. Furthermore, it remains to be understood whether some of the T-cell-priming APCs in the MLN are actually AMs, the AMs that have differentiated into DCs, or *M.tb-*loaded neutrophils.

### 3.3. Passive Transport of *M.tb* to the MLN

 In addition to the active transport of *M.tb/M.tb *antigen to the MLN by migratory DC or AM populations, it has been suggested that the passive transport of antigen could be accomplished via the lymphatic drainage of the lung. Whether the *M.tb *organism actively utilizes this system to mediate its “cell-free” dissemination from the lung to the MLN is unknown. It remains plausible that discrepancy in the time of bacterial arrival to the MLN and the time of T-cell priming could be attributed to cell-free transport of *M.tb*. Regardless of how *M.tb* arrives in the lymph node, the appropriate activation of naïve T-cells depends on the interaction between the antigen-loaded DCs and their cognate naïve T-cell. Critical to this interaction is the expression of sufficient levels of costimulatory molecules, a high density of MHC loaded with the cognate antigen, and the production of polarizing cytokines. The inflammatory microenvironment during the acquisition of antigen plays an integral role in the maturation of DC populations and subsequent T-cell priming.

### 3.4. Mechanisms of Delayed T-Cell Priming

 It has long been recognized that *M.tb* utilizes the induction of IL-10 as a means to suppress effector cell function. Specifically, it has been demonstrated that infected macrophage and DC populations can produce high levels of IL-10 in response to live, but not heat-killed, *M.tb *[64, 65]. It has been demonstrated that upon infection, *M.tb* employs multiple secreted virulence factors to subvert host recognition, many of which actively impair antigen processing and loading, and the surface expression of MHC class II [66–68]. Most notably, the 19 kDa protein secreted by *M.tb* has been shown to inhibit the activation of several genes involved in antigen presentation, including the downregulation of MHC class II, HLA-DM, and CIITA [66, 67, 69]. In addition to impairing antigen processing, *M.tb*'s major cell wall component, cord factor (trehalose 6,6′-dimycolate) has been shown to significantly impair the upregulation and appropriate expression of costimulatory molecules such as CD28 [70]. Together, these impairments are thought to alter or delay T-cell priming [71]. Furthermore, the expression of high levels of IL-10 results in the preferential induction of an early T regulatory cell population that serves to delay the initiation of protective type 1 immune responses [72].

While virulence has long been considered an underlying mechanism for the relative delay in T-cell priming, the evidence from others and us suggests that this delay is independent of the relative virulence of the mycobacterium itself, as delayed T-cell priming has also been observed following infection with attenuated strains of *M.tb* or BCG [40, 73]. Rather, it is possible that delayed T-cell priming is due to factors that are inherent to slow-growing mycobacterial species. Many species of pathogenic and nonpathogenic mycobacteria exist in nature. It has been observed that “pathogenic” *mycobacterial* spp., such as *M.tb*, BCG, and *M. avium,* replicate slowly, lead to delayed immune activation, and are capable of persistent disease. On the other hand, “nonpathogenic” *mycobacterial *spp., such as *M. smegmatis* or *M. fortuitum, *replicate quickly, evoke a fast T-cell response, and are rapidly cleared [74, 75]. Slow-growing *mycobacteria* such as *M.tb* have developed many strategies to remain immunologically inert, fundamental to which are unique modifications to its cell wall [12, 76]. Compared with nonpathogenic mycobacteria, the capping of lipoarabinomannan (LAM), a key cell wall component, is unique. Following further examination, it was revealed that the cell wall of all pathogenic, slow-growing mycobacteria contained mannose capped LAM (manLAM). Further, it was shown that all nonpathogenic, fast-growing mycobacterial cell walls contained uncapped or phosphor-*myo*-inositol- (PI-) capped LAM [75, 77–81]. The mannose capping of LAM has been shown to facilitate many immunological events, including phagocytosis by the AM [20], impaired cytokine and chemokine production [27], delayed phago-lysosome fusion [82], and suppressed DC activation [83]. It is now commonly accepted that manLAM is highly immunosuppressive, while uncapped or PI-capped LAM is strongly immunogenic [75, 80, 84]. While it represents an interesting postulate, the role that immunesuppressive manLAM plays in delayed T-cell priming following *M.tb* infection remains unknown.

The ability of *M.tb* to survive in the cell relies heavily on its unique ability to subvert the innate and adaptive immune systems. Its unique cell wall structure composed of lipids and glycoproteins mediates its survival in the phagosome, primarily through arresting fusion with the lysosome. One of the major components of the cell wall is mannose-capped LAM which is thought to be critical to arresting phagolysosome fusion [85, 86]. The ability of LAM to arrest phagosome fusion relies on its ability to prevent the phosphorylation of phosphatidylinositol 3-phosphate (PI3P), a required step in the conversion of an early phagosome to a late phagosome [85]. The ability of LAM to prevent the phosphorylation of PI3P is mainly attributed to its ability to prevent the cellular influx of Ca^2+^, a required step in the activation of phosphatidylinositol kinase (PI3K) [85]. In addition to LAM, the activation of PI3P is further prevented by SapM, a secreted PI3P-phosphatase, further ensuring that the phagosome is arrested at the early stage [85]. In addition to preventing phagosome maturation, *M.tb* encodes a number of proteins directed at survival in an activated phagolysosome. The ability to combat reactive nitrogen intermediates and reactive oxygen species is critical to *M.tb*'s survival following the induction of adaptive immunity and correlates with strain virulence [86, 87]. *M.tb* encodes two superoxide dismutases, sodA and sodC, which catalyse the conversion of superoxide anions to H_2_O_2,_ and a catalase-peroxidase katG to combat the increased levels of H_2_O_2_ [87]. Furthermore, *M.tb* encodes a combined NADH-dependent peroxidase and peroxynitritesreductases which is composed of four protein components; an alkyhydroperoxidereductase, a thioredoxin-related oxidoreductase, a dihydrolipoamideacyltransferase, and a lipoamide dehydrogenase [87]. These four components function to detoxify both RNI and ROS and protect *M.tb* from the harsh environment of an activated phagolysosome, limiting the availability of *M.tb* antigen [87]. The ability of *M.tb* to survive within the APC, coupled with its slow replication rate, functions to limit the amount of antigen available to prime required T-cell responses. To support this notion, we have noted that a 10-fold increase in the infectious dose of mycobacteria could enhance the arrival of bacteria to the MLN and accelerate T-cell priming [unpublished data], indicating that the antigen load in the lung may be responsible for the delayed T-cell priming seen in the MLN.

Alterations to the inflammatory microenvironment can significantly impair the ability of responding DCs to appropriately initiate adaptive immune responses that are central to the control of *M.tb* infection. The ability of *M.tb* to live intracellularly shields it from the host's humoral response. Thus, controlling bacterial dissemination and curtailing its replication is largely the responsibility of activated T-cells subsets. Owing to the intracellular and intraphagosomal nature of *M.tb*, antigen loading is primarily through the MHC class II pathway. As such, the dominant T-cell subset induced is that of a CD4^+^IFN-*γ* ^+^. Additionally, mycobacterial antigen is loaded on the MHC class I pathway by either cross-presentation mediated by the uptake of apoptotic bodies or phagosomal escape, allowing for the priming of antigen-specific CD8 T-cell [88–91]. Studies using MHC class II and class I deficient mice have demonstrated the relative contribution of CD4 and CD8 T-cells to *M.tb* immunity. While a deficiency in MHC class I has a limited impairment on bacterial control, deficiency in MHC class II results in extensive impairment, signifying the relatively greater importance of CD4 T-cells [90]. Given the central role of IFN-*γ* in macrophage activation and nitric oxide production, a greater impairment is seen in iNOS-deficient mice then in either MHC class I or II deficient mice, thus indicating that IFN-*γ* from both type I CD4 and CD8 T-cells plays a critical role in protection [90]. While a type 1 immune response eventually ensues, the delayed T-cell priming by early immune evasion strategies employed by *M.tb* provides a critical window for *M.tb* to grow completely unchecked in the lung.

## 4. Effector T-Cell Recruitment to the Lungs

As expected, delayed T-cell priming in the lymph nodes of *M.tb*-infected animals results in delayed arrival of effector T-cells at the lung, the primary site of infection (Figure 2). This permits *M.tb* to increase logarithmically within the lungs of the host for approximately 20 days, thus establishing a robust “foothold” prior to the arrival and abundant presence of antigen-specific T-cells at the site of infection [92–94]. The mass arrival of T-cells to the lungs occurring between 18 and 20 days postinfection is associated with the ultimate plateau of bacterial growth [40, 92, 94, 95].

### 4.1. Recruitment of T-Cells to the Different Lung Compartments

 The lung can be divided into two main compartments: the interstitial tissue existing between the alveoli, and the mucosal surface of the lung known as the airway lumen (Figure 2). While the timing of T-cell priming in the MLN ultimately determines the kinetics of effector T-cell migration to the infected lung, there is growing evidence to suggest that the coordinated upregulation of several molecules is essential to the homing of T-cells to the lung. Specifically, the most recent focus has been on the kinetics of chemokine production as well as the coordinated upregulation of specific adhesion molecules. Both the expression of integrins on T-cells and their respective addressin molecules on the vascular endothelium and alveolar epithelium are essential to appropriate recruitment of effector T-cells to lung. The differential upregulation of these molecules dictates whether a T-cell traffics to the lung interstitium or airway lumen and can dramatically effect bacterial control. While it is believed that T-cell trafficking to the airway lumen is a required process for the control of *M.tb *[96], little work has been done to understand the differential inflammatory signals required to recruit T-cells into the airway lumen. Based on this, most studies have focused on the molecules required for recruiting T-cells into the lung as a whole rather than the differential lung compartments.

### 4.2. Critical Chemotactic Molecules

 It has been demonstrated that CCL5 (RANTES) dramatically increases between day 10 and day 20 post-*M.tb* infection [97]. However, the specific role of this chemokine in T-cell homing to the lungs has only recently been elucidated. Through the use of CCL5 knockout (KO) mice, Vesosky et al. [98] have shown that CCL5 is critically required for the early recruitment of CCR5-expressing CD4 T-cells to the lung in *M.tb*-infected mice. The delay in effector T-cell recruitment in CCL5 KO mice caused a significant reduction in IFN-*γ* production and impaired granuloma formation, resulting in significantly higher bacterial burden within the lungs of these animals when compared to wild type controls [98].

Best known for their critical role in DC homing to the MLN, CCL19 (MIP-3*β*), and CCL21 have been recently shown to be essential in the trafficking of IFN-*γ* ^+^ T-cells from the MLN to the lungs of *M.tb*, infected mice. In the study conducted by Khader [99], CCL19 was shown to increase in the lungs of infected mice between 15 and 18 days, correlating with the arrival of effector T-cells and the initiation of granuloma formation. Mice deficient in CCL19 and 21 showed significantly impaired CD4^+^  IFN-*γ* ^+^ T-cell kinetics to the lungs prior to day 30 post-infection [99]. The blunted T-cell recruitment in the lungs of CCL19/CCL21-deficient mice resulted in delayed IFN-*γ* and iNOS production, macrophage activation, and bacterial control [99]. This leads to severely impaired granuloma formation and increased bacterial loads for at least 80 days postinfection, indicating the critical role of timely T-cell trafficking to the lung [99].

### 4.3. Adhesion Molecules

 In addition to chemokine expression within the lung, several studies have focused on identifying the required adhesion molecules and specific integrins which mediate the entry of effector T-cells into the lung. Vascular cell adhesion molecule 1 (VCAM-1) expression is upregulated in *M.tb* infected lungs by day 21 and is associated with the recruitment of the majority of IFN-*γ*-producing T-cells [100]. The preferential expression of *α*_4_ *β*_1_ or *α*_4_ *β*_7_ on activated CD4 T-cells makes VCAM-1 essential to efficient recruitment of T-cells into lung [100]. Furthermore, depletion of either *α*_4_ or *α*_4_ *β*_7_ results in a significant decrease in the number of lymphocytes within the lung, the consequence of which manifests in granulocyte-dominated granulomas consisting of disorganized infiltrates and heightened necrosis [100]. Similar defects in granuloma formation were seen in the lungs of mice deficient CD11a/18, where a 3-4 fold reduction in the number of antigen-specific T-cells recruited resulted in increased neutrophilia, necrotic foci, and poorly formed granulomas [101]. It is therefore apparent that the timing of effector T-cell trafficking into the lungs following *M.tb* infection is critical to the establishment of granuloma formation as well as appropriate bacterial control.

## 5. Effector Functions of Recruited T-Cells in the Lung

Upon arrival in the lung, effector T-cells are thought to mediate protection by two primary mechanisms: (1) the activation of infected macrophages to produce antimicrobial substances, and (2) the physical segregation of infected cells to granuloma structures. While in 90% of infected humans these methods allow for the host to control *M.tb* dissemination and achieve latency, rarely is the bacteria cleared.

### 5.1. Macrophage Activation

 The airway lumen is largely considered the primary site of infection. With effector T-cells being recruited to the airway lumen, T-cell-derived IFN-*γ* activates infected AMs to mediate enhanced phagosome lysosome maturation, upregulation of MHC class II loading, and the induction of highly toxic antimicrobial substances. The increase in MHC class II expression allows infected macrophages to be targeted by Th1 CD4 T-cells, and either activated to kill internalized bacteria or removed by Fas/FasL or TNF-directed apoptosis [2, 102–105]. Following IFN-*γ* mediated activation, the infected macrophage generates both reactive oxygen substances (ROSs) and reactive nitrogen intermediates (RNIs) [106, 107]. Although the generation of ROS, such as H_2_O_2_, has been demonstrated following *M.tb *infection, it is believed that the major mediator of antimycobacterial action is through the generation of RNI, specifically nitric oxide (NO) by the inducible nitric oxide system iNOS-NOS2 [106, 107]. Limited resistance to RNIs is a common feature of mycobacteria, with the most virulent strains such as *M.tb *and *M.bovis* being almost completely resistant [106, 107]. Although sterile clearance is never achieved, the activation of an infected macrophages is thought to be strongly bacteriostatic, facilitating the persistence of *M.tb* within the host [107]. The key role of IFN-*γ* in this process is without question and was conclusively shown with murine IFN-*γ*-deficiency models [108]. In the absence of IFN-*γ*, mice fail to upregulate NOS2 and are unable to control bacterial dissemination, succumbing to the infection within the first few weeks [108]. Despite the unprecedented role of IFN-*γ*, it cannot function alone. IL-12 was shown to be essential to the optimal induction of both NO and TNF [109]. Other studies have demonstrated an essential role for TNF and it is now believed that IL-12, IFN-*γ*, and TNF must be present for optimal NO production [103, 105].

### 5.2. Granuloma Formation

 The induction of what is termed the “immune” granuloma is the hallmark of immune mediated control and is thought to represent the primary mechanism of long-term protection. The formation of the “immune” granuloma is a very operose process, which follows the arrival of the adaptive immune cells to the lung, an event not normally seen until 2-3 weeks postinfection as aforementioned [60]. The “immune” granuloma induces a number of defined histopathological changes to the innate granuloma structure. The innate granuloma is further fortified by the arrival of effector T-cells, and the ability of infected macrophages to kill internalized bacteria is enhanced by the release of IFN-*γ* [99]. The addition of effector T-cells to the granuloma produces what is termed the lymphocytic cuff, where entering lymphocytes surround the infected macrophage populations forming a barrier, adjoining with the focal infection [2, 89, 104, 110–112]. The formation of the lymphocytic cuff and the ensuing production of inflammatory mediators results in defined structural changes to the partitioned macrophage populations. Two major morphological changes occur in the infected macrophage populations, within the type I granuloma: first, the induction of an epithelial-like appearance, producing epithelioid macrophages, and second, the fusion of macrophage populations to form multinucleated giant cell populations [2, 89, 104, 110–112]. The arrival of effector T-cells and the formation of an “immune” granuloma correlates with the cessation of bacterial growth and a plateau is reached [2, 89, 104, 110–112]. Type 1 cytokines including IL-12, IFN*γ*, and TNF*α* required for macrophage activation are also essential to granuloma formation [113] or the maintenance of granuloma [114].

### 5.3. Granuloma as a Symbiotic Microenvironment for Mycobacterial Persistence

 Recently, many groups have begun to compartmentalize the granuloma away from the lung parenchyma and airway lumen. Mounting evidence from us and others suggests that the granuloma represents an immunologically suppressed or dampened zone that the mycobacterium prefers to dwell in [115–117]. The immunological suppression of the granuloma was determined to be due to high levels of IL-10, which functioned to suppress both APC and T-cell populations within the granuloma [116]. Granuloma-derived IL-10 suppresses many effector functions of the resident APC populations. Within the granuloma, APCs show an impaired ability to phagocytose mycobacteria, drive T-cell priming, and produce nitric oxide [116]. Granuloma resident T-cell populations are also phenotypically distinct, having a regulatory-like function and producing high levels of IL-10 and low levels of IFN-*γ* [116]. Removing IL-10 from the model was shown to reverse the suppression on the APC and T-cell populations in the granuloma and overall better bacterial control was achieved [116]. Thus, the renewed knowledge of mycobacterial granuloma biology helps us further understand why the host immune system can hardly eliminate infection and often the latency is the best outcome.

## 6. Role of Airway Luminal T-Cells (ALTs) in Anti-TB Immunity

Primary mycobacterial infection studies have demonstrated that the delayed effector response in the lung is a direct result of retarded T-cell priming in the MLN. As described above, the lung can be divided into two main compartments: the interstitial tissue residing in between the alveoli, and the mucosal surface of the lung known as the airway lumen. Although the current literature describes the kinetics of effector T-cell responses in the whole lung, the role of ALTs in the airway lumen have been largely neglected to date [96, 118]. Recently, our group and others have begun to characterize the T-cell kinetics in these two lung tissue compartments. Despite the earlier arrival of effector T-cells in the lung interstitium, following *M.tb *infection, it is the arrival of ALTs to the airway lumen that is associated with the plateau of bacteria replication [119], (Figure 2). Inspired by the plethora of TB vaccine studies understanding the role of T-cells in the differential lung compartments, this is the first study to characterize the T-cell responses in both the lung interstitium and airway lumen following a primary challenge.

### 6.1. Mucosal versus Parenteral Vaccination Against *M.tb*

 Over the past decade, the development of novel TB vaccine candidates has produced a wealth of knowledge on the ways in which TB vaccination can be improved. Arguably the most significant finding has been the repeated observation that vaccinating mucosally provides enhanced protection over parenteral immunization against pulmonary *M.tb* infection. In particular, work from our laboratory has demonstrated that a recombinant adenoviral vector expressing *M.tb* antigen 85a (AdAg85a) when delivered intranasally was seen to provide remarkably enhanced protection against virulent *M.tb* challenge compared to intramuscular administration [120]. Furthermore, intranasal vaccination was able to provide superior protection over the “Gold Standard” subcutaneous BCG immunization, with at least an additional log reduction in bacterial burden following *M.tb* infection [120]. However, the AdAg85a work does not stand alone, as several others have reported that mucosal immunization provides greater protection against *M.tb* infection compared to parenteral vaccination [121, 122].

### 6.2. Requirement of ALTs

 Investigation into the mechanism by which this enhanced protection is achieved revealed that mucosal but not parental vaccination with AdAg85a resulted in the generation of a population of antigen-specific T-cells that reside within the airway lumen [123]. The failure of parenteral vaccination to generate a population of ALTs can be largely attributed to the lack of an inflammatory response generated in the airway [123]. This defect is particularly evident in experiments where the delivery of soluble mycobacterial antigens to the airway elicits a potent inflammatory response characterized by heightened levels of TNF, MIP-1*α*, MCP-1, and IP-10 [124, 125]. The production of these inflammatory mediators functions to draw in peripherally primed T-cells, enhancing protection to a level comparable to that of mucosally vaccinated animals [124, 125]. The neutralization of IP-10 or MIP-1*α* at the time of soluble protein delivery significantly impaired the recruitment of peripherally primed T-cells into the airway, impairing protection, thereby demonstrating the critical role of these chemokines in populating this compartment [124, 125]. Specifically, ALTs were found to be critical for protection as they are capable of responding quickly upon *M.tb* infection, eliminating the delay of effector responses [124, 125]. These findings indicate that both the timing and geographic localization of T-cell responses is critical to the efficiency of bacterial control. Following primary *M.tb* infection, it is essential to understand the role that the various immune molecules play in the recruitment of T-cells both to the lung interstitium, and most importantly to the airway lumen. Such knowledge will provide further insight into the mechanisms of delayed or impaired T-cell trafficking to the lung, and thus provide ways by which protection against *M.tb* can be enhanced.

## 7. Concluding Remarks

Understanding the mechanisms of delayed T-cell priming in the MLN and delayed effector T-cell recruitment to the lung interstitium and airway lumen following pulmonary *M.tb* infection is critical for us to develop effective anti-TB vaccine and therapeutic strategies. The current vaccine initiatives are significantly hampered by our limited knowledge of what a protective immune correlate looks like as following infection *M.tb is rarely cleared*. Although much progress has been made in understanding the kinetics of T-cell priming following *M.tb*, the mechanisms underlying this delay are only beginning to be understood. Given the intimate relationship between the host and bacteria, it is of paramount importance for us to further dissect the differential contributions of both the host and pathogen to the relative delay in T-cell priming and recruitment, and their impact on bacterial control.
